# Recent insights into the biological functions of baicalin

**DOI:** 10.17179/excli2022-5184

**Published:** 2022-08-01

**Authors:** Priscilla Nadalin, Jae Kwang Kim, Tae Won Kim, Sang Un Park

**Affiliations:** 1 Department of Crop Science, Chungnam National University, 99 Daehak-ro, Yuseong-gu, Daejeon, 34134, Korea; 2 Division of Life Sciences and Convergence Research Center for Insect Vectors, College of Life Sciences and Bioengineering, Incheon National University, Incheon 22012, Korea; 3 College of Veterinary Medicine, Chungnam National University, 99 Daehak-ro, Yuseong-gu, Daejeon 34134, Korea

## ⁯

Flavonoids are molecules possessing a 15-carbon skeleton structure consisting of two aromatic ring systems (A and B rings) and a heterocyclic ring (C). This carbon backbone can be shortened to C6-C3-C6 (Kumar and Pandey, 2013[20]). Flavonoids can be sorted into different subgroups, based on the degree of unsaturation and substitution patterns, consisting of anthocyanins, catechins, chalcone flavanols, flavanones, flavones, flavanonols, flavonols, and isoflavonoids (Santos-Buelga and Feliciano, 2017[38]).

Flavones (from the Latin term *flavus *meaning "yellow") belong to the group of flavonoids that share a 2-phenylchromen-4-one (2-phenyl-1-benzopyran-4-one) backbone, and are secondary metabolites commonly present in plants and fungi, and are naturally yellow-colored (Panche et al., 2016[34]; Kumar and Pandey, 2013[20]).

Baicalin, a flavone glycoside, is one of the most important bioactive compounds in *Scutellaria radix* (the dry raw root of *Scutellaria baicalensis*), which is among the 50 essential herbs used in traditional Chinese medicine (Liang et al., 2017[26]). Baicalin is a 7-*O*-glucuronide derivative of baicalein, and its synthesis is catalyzed by baicalein 7-*O*-glucuronosyltransferase in *S. baicalensis *(Nagashima et al., 2000[32]).

It has been reported effective in exerting several pharmacological activities, namely antibacterial, antiviral, anticancer, anticonvulsant, anti-inflammatory, antioxidant, hepatoprotective, and neuroprotective effects (Cui et al., 2022[5]; Wang et al., 2022[51]; Li et al., 2021[24]; Pan et al., 2021[33]). The pharmacological properties of baicalin are attributed to its ability to scavenge reactive oxygen species (ROS) and interact with several signaling molecules related to apoptosis, autophagy, cell cycle, cytoprotection, inflammation, and mitochondrial dynamics (Hu et al., 2022[14]). The therapeutic potential of baicalin warrants further studies as a natural treatment for various human diseases. Here, we report current findings on baicalin's biological properties and pharmacological activities (Table 1(Tab. 1); References in Table 1: Ai et al., 2022[1]; Cai et al., 2022[2]; Changle et al., 2022[3]; Chen et al., 2021[4]; Dou et al., 2020[6]; Fan et al., 2021[8], 2022[7]; Fang et al., 2020[9], 2022[10]; Fu et al., 2020[11]; Hao et al., 2021[12]; He et al., 2022[13]; Huang et al., 2020[15]; Ishfaq et al., 2021[16]; Ji et al., 2022[17]; Jia et al., 2021[18]; Kong et al., 2021[19]; Kunimatsu et al., 2022[21]; Li and Tang, 2021[23]; Li et al., 2020[25], 2022[22]; Lin et al., 2020[27], 2022[28]; Liu et al., 2020[29]; Ma et al., 2021[30]; Miao et al., 2021[31]; Paudel and Kim, 2020[35]; Peng et al., 2021[36]; Rizzo et al., 2021[37]; Shah et al., 2020[39]; Shehatta et al., 2022[40]; Shi et al., 2020[41]; Song et al., 2022[42]; Su et al., 2021[43]; Sui et al., 2021[44]; Sun et al., 2021[46], 2022[45]; Tan et al., 2021[47]; Tsai et al., 2021[48]; Wang et al., 2020[50], 2021[49]; Wu et al., 2020[53], 2021[52]; Xiang et al., 2021[54]; Xiao et al., 2021[55]; Xu et al., 2020[56]; Yang et al., 2020[57], 2022[58]; Yoshida et al., 2021[59]; You et al., 2022[60]; Yu et al., 2022[61]; Zeng et al., 2020[62]; Zhang et al., 2020[64], 2021[63]; Zhao et al., 2021[65]; Zhen et al., 2021[66]; Zou et al., 2021[67]). 

## Notes

Priscilla Nadalin and Jae Kwang Kim contributed equally as first author.

## Declaration

### Acknowledgments

This research was supported by the Bio & Medical Technology Development Program of the National Research Foundation (NRF) & funded by the Korean government (MSIT) (No. 2022M3E5E6018649). First author would like to express her deepest gratitude to the Korean Government (GKS-G-2020-393) for providing funding for her PhD studies.

### Conflict of interest

The authors declare no conflict of interest.

## Figures and Tables

**Table 1:**
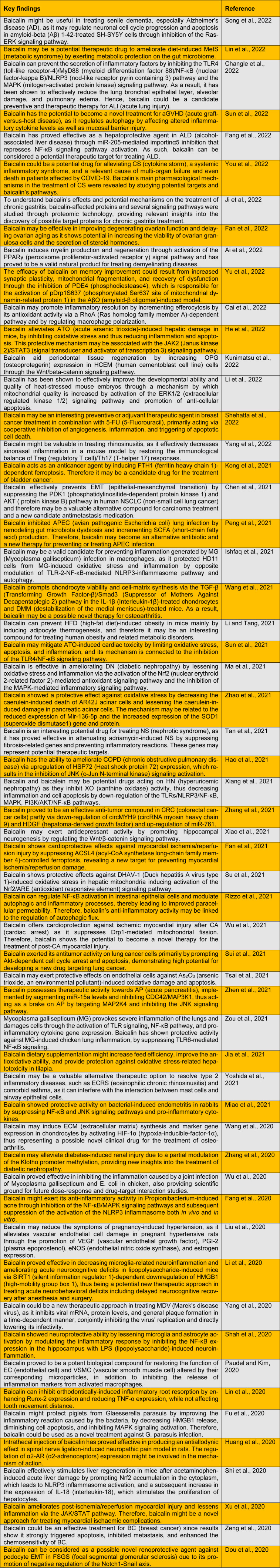
Recent studies on the biological and pharmacological activities of baicalin
